# Development and Application of a System Based on Artificial Intelligence for Transcatheter Aortic Prosthesis Selection

**DOI:** 10.21470/1678-9741-2018-0072

**Published:** 2018

**Authors:** Álvaro M. Rösler, Jonathan Fraportti, Pedro Nectoux, Gabriel Constantin, Sílvio Cazella, Mauro Ricardo Pontes Nunes, Fernando A. Lucchese

**Affiliations:** 1 Hospital São Francisco - Santa Casa de Misericórdia de Porto Alegre, Porto Alegre, RS, Brazil.; 2 Universidade Federal de Ciências da Saúde de Porto Alegre - UFCSPA, Porto Alegre, RS, Brazil.

**Keywords:** Expert System, Artificial Intelligence, Aortic Valve Stenosis, Transcatheter Aortic Valve Replacement

## Abstract

**Introduction:**

The interest in Expert systems has increased in the medical area. Some of them are employed even for diagnosis. With the variability of transcatheter prostheses, the most appropriate choice can be complex. This scenario reveals an enabling environment for the use of an Expert system. The goal of the study was to develop an Expert system based on artificial intelligence for supporting the transcatheter aortic prosthesis selection.

**Methods:**

The system was developed on Expert SINTA. The rules were created according to anatomical parameters indicated by the manufacturing company. Annular aortic diameter, aortic area, aortic perimeter, ascending aorta diameter and Valsalva sinus diameter were considered. After performing system accuracy tests, it was applied in a retrospective cohort of 22 patients with submitted to the CoreValve prosthesis implantation. Then, the system indications were compared to the real heart team decisions.

**Results:**

For 10 (45.4%) of the 22 patients there was no concordance between the Expert system and the heart team. In all cases with discordance, the software was right in the indication. Then, the patients were stratified in two groups (same indication *vs*. divergent indication). The baseline characteristics did not show any significant difference. Mortality, stroke, acute myocardial infarction, atrial fibrillation, atrioventricular block, aortic regurgitation and prosthesis leak did not present differences. Therefore, the maximum aortic gradient in the post-procedure period was higher in the Divergent Indication group (23.9 mmHg *vs*. 11.9 mmHg, *P*=0.03), and the mean aortic gradient showed a similar trend.

**Conclusion:**

The utilization of the Expert system was accurate, showing good potential in the support of medical decision. Patients with divergent indication presented high post-procedure aortic gradients and, even without clinical repercussion, these parameters, when elevated, can lead to early prosthesis dysfunction and the necessity of reoperation.

**Table:** 

Abbreviations, acronyms & symbols	
TAVI	= Transcatheter aortic valve implantation

## INTRODUCTION

Interest in computational Expert systems has increased greatly in the medical area in the last decade. The main function of these systems is to allow, through storage, sequencing and use of specialized knowledge solving problems using softwares. Some of these systems are already used successfully to provide diagnostic support and promoting prevention actions in the healthcare area^[1-3]^.

In addition, these softwares are developed with a knowledge data base equivalent to that of Experts in a very specific area. They allow non-specialists to solve problems, increasing agility during the consultations and are also less susceptible to errors^[4]^. Among the different types of Expert systems, the rule-based model is usually a more adequate representation of knowledge and provide a greater transparency, given the simplicity with which rules are usually exposed and how the knowledge is organized. In this way, Expert systems that follow rule models are usually quite accessible and provide rapid responses to complex problems^[5,6]^.

The number of cardiac valvular procedures performed through catheter is increasing around the world and with the variability of available transcatheter aortic prostheses, the most appropriate choice of prosthesis can become a very complex process that requires the participation of several professionals. The choice of prosthesis for a patient affected by aortic stenosis is based on a series of anatomical features of the aortic valve annulus, such as: diameter, area and perimeter. The diameters of the ascending aorta and the Valsalva sinus are also taken into account. These measurements are obtained by angiotomographic examination and are mandatory for prosthesis selection^[7,8]^.

This scenario reveals an environment which allow the use of specialized software. Considering that a series of anatomical data needs the involvement of different professionals with different levels of knowledge in the area, the chance of dubious decisions is considerable. Therefore, the main objective was to develop an Expert system to support the selection of the most appropriate transcatheter aortic prosthesis and to compare the accuracy of the indications provided by the system with decisions already made by a heart team in a retrospective series of patients submitted to transcatheter aortic valve implantation (TAVI).

## METHODS

**Fig. 1:**
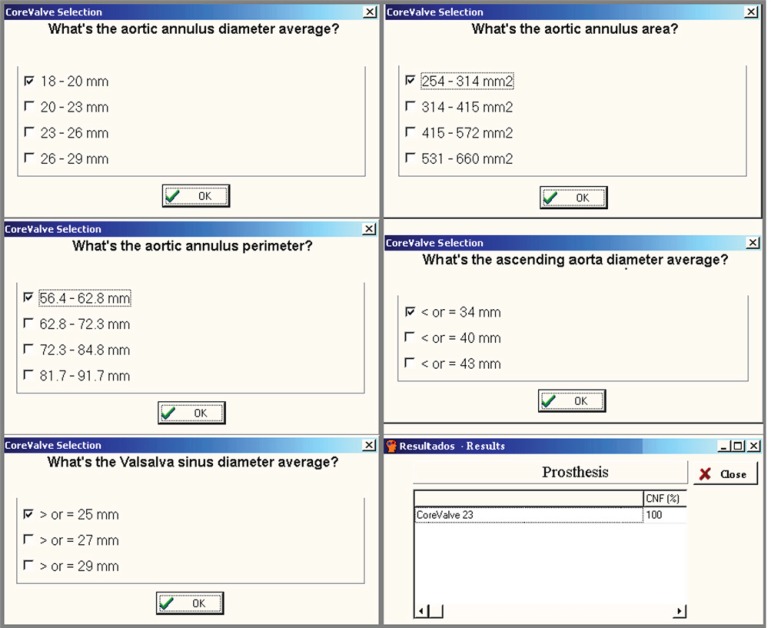
Expert system for the selection of the CoreValve transcatheter prosthesis.

The Expert system for the selection of CoreValve transcatheter prosthesis (Figure 1) was developed by the Cardiovascular Surgery Research Center at Hospital São Francisco - Santa Casa de Misericórdia de Porto Alegre through the Expert SINTA platform^[4]^. This platform enables professionals with limited knowledge in the informatics area to develop their own Expert systems according to their needs and specific problems. For now, the Expert system is compatible with Windows operational systems and can be requested to the study author by e-mail.

Initially, a thorough bibliographical review was performed to accurately select the variables used to indicate the most appropriate size of the CoreValve prosthesis (Medtronic(r) Minneapolis, MN, USA). Based on the data provided by the manufacturer, five mandatory variables were identified for the appropriate choice of prosthesis: aortic annulus mean diameter, aortic annulus area, aortic annulus perimeter, mean ascending aortic diameter, mean diameter of the aorta and of Valsalva sinus. The choice of prosthesis should follow the standards described in Table 1. All measurements were obtained by the angiotomographic images according to previous guidelines of cardiovascular radiology^[9]^. These indication patterns were the basis for elaborating the rules attached in the Expert system.

**Table 1:** CoreValve variables and patterns used to select the ideal prosthesis.

Prosthesis number	Annulus diameter	Annulus area	Annulus perimeter	Ascending aorta	Valsalva sinus
23	18 - 20 mm	254 - 314 mm^2^	56.4 - 62.8 mm	≤ 34 mm	≥ 25 mm
26	20 - 23 mm	314 - 415 mm^2^	62.8 - 72.3 mm	≤ 40 mm	≥ 27 mm
29	23 - 26 mm	415 - 572 mm^2^	72.3 - 84.8 mm	≤ 43 mm	≥ 29 mm
31	26 - 29 mm	531 - 660 mm^2^	81.7 - 91.7 mm	≤ 43 mm	≥ 29 mm

After the creation of the rules, user interface formatting and inclusion of explanatory information, system tests were performed to confirm the accuracy of the indications. Fifty tests were performed, which reached 100% accuracy according to the criteria standards indicated by the prosthesis manufacturer. These results were confirmed by three specialized researchers in cardiovascular imaging and TAVI. After the accuracy tests, the Expert system was used to review size indications of CoreValve prosthesis in a retrospective cohort of 22 patients who underwent transcatheter aortic prosthesis implantation with prosthesis select based on the standard medical decision and without the support of any software.

Then, the indications obtained through the Expert system for patients in the cohort were compared with the previous decisions of the heart team at the time. The São Francisco Hospital maintains a permanent database of the patients who undergo transcatheter valve procedure and from this, the data pertinent to the study was obtained and used to evaluate the prosthesis size by the Expert system. The same informations considered by the medical team at the time were applied in the software evaluation, without any differences or modifications in the previous data.

The data was tabulated, coded and analyzed using the SPSS IBM V23 Statistical Software. Descriptive inference was carried out through the verification of frequencies and measures of central tendency and dispersion for numerical variables. Data analysis included statistical tests for qualitative variables (Chi-square, Fisher's test) and for quantitative variables (test T for independent variables). The variables were submitted to the normality test of Komolgorov-Smirnov. The significance level considered was 5%. All ethics requirements were complied according the Brazilian regulations and the Hospital's demands.

## RESULTS

Between November 2009 and June 2015, 22 transcatheter aortic prosthesis implants were performed using the CoreValve prosthesis at São Francisco Hospital. All patients in this period were included in the study. The average patient age was 82.3 years, and 22.7% were female.

In 10 (45.4%) of the 22 patients there was no agreement between the Expert system and the heart team indications. The tests were repeated and enhanced the accuracy of the results. The accuracy of the Expert system was 100% and the accuracy of the heart team indication was 54.5% (Table 2). Based on these results, the patients were stratified in two groups: same indication (12 patients) and divergent indication (10 patients) between the Expert system and the heart team. In the analysis of the baseline characteristics, no significant difference between the groups was found (Table 3).

**Table 2:** Prosthesis aortic sizes by the industry, Expert system and previous medical decision.

Patient code	Industry recommendation	Expert System indication	Medical decision	Same indication
CV-01	29	29	26	No
CV-02	31	31	29	No
CV-03	No prosthesis available*	No prosthesis available*	29	No
CV-04	26	26	29	No
CV-05	29	29	29	Yes
CV-06	29	29	26	No
CV-07	29	29	29	Yes
CV-08	31	31	29	No
CV-09	31	31	29	No
CV-10	31	31	31	Yes
CV-11	29	29	29	Yes
CV-12	29	29	31	No
CV-13	31	31	29	No
CV-14	29	29	29	Yes
CV-15	29	29	29	Yes
CV-16	29	29	29	Yes
CV-17	31	31	31	Yes
CV-18	29	29	29	No
CV-19	31	31	29	No
CV-20	31	31	31	Yes
CV-21	29	29	29	Yes
CV-22	31	31	31	Yes

* Not compatible with any of the available sizes of the CoreValve prosthesis.

**Table 3:** Comparison of the patients' baseline characteristics according the group of study.

Variables	Same indication (n = 12)	Divergent indication (n = 10)	*P*
Female gender	3 (25%)	2 (20%)	1.000
Age (years)	84.8±3.4	79.9±7.2	0.073
Body mass index (kg/m^2^)	24.3±3.7	25.2±2.7	0.556
Previous cardiovascular surgery	4 (33.3%)	2 (20%)	0.646
Systemic arterial hypertension	11 (91.7%)	9 (90%)	1.000
Diabetes mellitus	3 (25%)	1 (10%)	0.594
Chronic or acute renal injury	5 (41.7%)	5 (50%)	1.000
Creatinine (mg/dl)	1.81±1.55	1.87±1.17	0.922
Myocardial infarction	__	1 (10%)	0.455
Stroke	2 (16.7%)	1 (10%)	1.000
Coronary disease	7 (58.3%)	2 (20%)	0.099
Tabagism	1 (8.3%)	1 (10%)	1.000
Obstructive pulmonary disease	1 (8.3%)	3 (30%)	0.293
Peripheral arteriopathy	__	1 (10%)	0.455
Atrial fibrillation	4 (33.3%)	5 (50%)	0.666
Atrioventricular block	__	2 (20%)	0.195
Ejection fraction of left ventricle (%)	58.7 ±19.1	56.2±16.1	0.742
Aortic medium gradient (mmHg)	45.1 ±15.7	46.5±17.4	0.844
Aortic maximum gradient (mmHg)	68.5 ±72.8	72.8±23.6	0.684
Aortic annulus area (cm^2^)	0.83 ±0.21	0.69±0.19	0.143
EuroSCORE I (%)	21.1 ±12.2	15.5±9.3	0.249
EuroSCORE II (%)	8.0 ± 6.3	4.7±2.8	0.147
STS Score (%)	7.5 ± 6.2	6.8±6.4	0.782
Observant Score (%)	4.8 ± 4.8	4.7±3.0	0.957

**Fig. 2:**
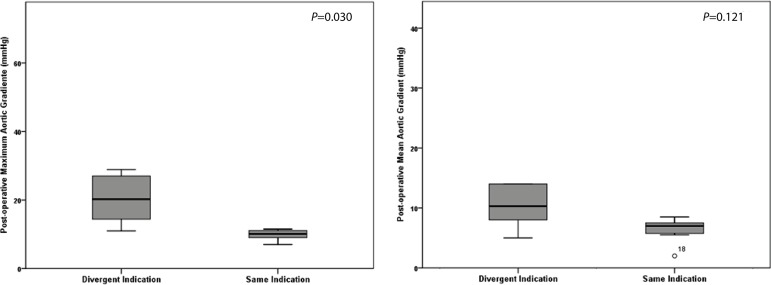
Boxplot graphs showing the difference in the post-operative aortic valve gradients according to prosthesis indication.

Clinical outcomes included postoperative incidence of the following adverse effects: mortality, stroke, acute myocardial infarction, atrial fibrillation, atrioventricular block, prosthesis regurgitation, prosthesis leakage, maximum and mean aortic gradient. Among the analyzed outcomes, the only one that presented significant difference between the groups was the maximum aortic gradient, which was higher in the group in which the decision of the heart team diverged from the choice of the Expert system and consequently from the standards indicated by the industry (Figure 2). The mean aortic gradient was also higher in the divergent group, although not a significant difference, a trend similar to the maximum aortic valve gradient (Table 4).

**Table 4:** Outcome incidences after transcatheter aortic valve procedure.

Outcomes	Same Indication (n=12)	Divergent indication (n=10)	*P*
Myocardial infarction	1 (8.3%)	__	1.000
Stroke	__	1 (10%)	0.455
Atrial fibrillation	2 (16.7%)	1 (10%)	1.000
Atrioventricular block	2 (16.7%)	1 (10%)	1.000
Aortic regurgitation	5 (41.7%)	3 (30%)	0.675
Aortic leak	4 (33.3%)	5 (50%)	0.666
Aortic valve mean gradient (mmHg)	7.7±4.7	12.6±9.1	0.121
Aortic valve maximum gradient (mmHg)	11.9±7.6	23.9±15.6	0.030
30-day mortality	2 (16.7%)	1 (10%)	1.000

## DISCUSSION

Expert systems are part of a subarea of artificial intelligence and are a form of knowledge-based system, aiming to provide conclusions on a specific topic. Therefore, Expert systems must have a knowledge database comprised of well-established facts and rules and be able to offer advice and solutions to certain problems in an assertive and rapid manner^[4,10]^.

Considering that almost half of the patients had a non-ideal aortic prosthesis implanted, even with the analysis of a multidisciplinary cardiology team, the use of an Expert system can generate a great impact in the planning of the transcatheter procedures, adding care quality through precision, providing safety in prosthesis selection. With the variability of factors and patterns that should be analyzed to select adequate transcatheter prosthesis, the present study area is very promising for the development of Expert systems for decision making support.

Our results showed a significant difference between the groups in the maximum aortic gradient. This important hemodynamic parameter was higher in the divergent group; in other words, the group with patients that received the non-ideal prosthesis by the heart team decision. The highest maximum aortic gradient in the patients in the divergent group has a very representative importance due to the long-term effect that this elevation can have on the prosthesis^[11,12]^. It has been known for many decades that conventional aortic prostheses made with very similar biological material to that of the transcatheter prostheses and that are submitted to high gradients tend to become dysfunctional sooner, resulting in the need for a new procedure for implanting a new prosthesis^[13-16]^.

Although only the maximum aortic gradient showed a significant difference, the mean aortic gradient presented a similar trend. These results are reinforced by the baseline characteristics of the patients, which in any of the clinical and preoperative variables presented some significant difference. This baseline patterns reduce the probability that possible confounding variables may affect the results of the postoperative gradients.

The accuracy of the specialist system and the divergence of the heart team's choices in relation to it call attention to some factors not yet established completely in the medical literature. Among these factors, the main ones are: eccentricity index and the degree of calcification of the aortic valve^[17-19]^. Another aspect of the case series that also rose attention was the number of cases with a divergent indication even with the participation of a proctor. The first eight cases were performed under the supervision of a specialist in transcatheter aortic implants with self-expanding prosthesis.

It is possible that in the next years these variables will be considered when choosing transcatheter prosthesis, but so far they have not been adopted by the industry. In addition, the occurrence of higher gradients in the divergent group demonstrates a tendency that the choice based on established industry standards may be safer.

## CONCLUSION

The use of the Expert system generated solid and reliable indications to the parameters of the manufacturer, demonstrating high accuracy in the valve selection. In addition, it was possible to identify that the choice of the medical team is influenced by subjective criteria not yet considered by the industry and without robust evidence in the medical literature. Patients in whom there was a divergence in the indication, between Expert system and heart team, presented high aortic gradients post-procedure. Even with no clinical repercussion and with the 22 procedures being successfully performed, these higher gradients can lead to early prosthesis dysfunction and, consequently, the need for reintervention in the patient.

**Table:** 

Authors' roles & responsibilities	
AMR	Conception and design of work; acquisition, analysis and interpretation of the data; drafting the work or revising it critically for important intellectual content; final approval of the version to be published
JF	Acquisition and interpretation of data; final approval of the version to be published
PN	Acquisition and analysis of data; final approval of the version to be published
GC	Acquisition and interpretation of data; final approval of the version to be published
SC	Final approval of the version to be published
MRPN	Final approval of the version to be published
FAL	Drafting the work or revising it critically for important intellectual content; final approval of the version to be published
